# The implementation of integrated care: the empirical validation of the Development Model for Integrated Care

**DOI:** 10.1186/1472-6963-11-177

**Published:** 2011-07-30

**Authors:** Mirella MN Minkman, Robbert P Vermeulen, Kees TB Ahaus, Robbert Huijsman

**Affiliations:** 1 Vilans, National Center of Excellence for Long-term care, PO Box 8228, 3503 RE Utrecht, The Netherlands; 2 Thorax Center, University Medical Center Groningen, University of Groningen, PO Box 30.001, 9700 RB, Groningen, The Netherlands; 3 University of Groningen, Faculty of Economics and Business, Research Center on Healthcare Organization & Innovation. University Medical Center Groningen Landleven 5, 9747 AD, Groningen, The Netherlands; 4 Erasmus University Rotterdam, Institute of Health Policy and Management, PO Box 1738, 3000 DR Rotterdam, The Netherlands

## Abstract

**Background:**

Integrated care is considered as a strategy to improve the delivery, efficiency, client outcomes and satisfaction rates of health care. To integrate the care from multiple providers into a coherent client-focused service, a large number of activities and agreements have to be implemented like streamlining information flows and patient transfers. The Development Model for Integrated care (DMIC) describes nine clusters containing in total 89 elements that contribute to the integration of care. We have empirically validated this model in practice by assessing the relevance, implementation and plans of the elements in three integrated care service settings in The Netherlands: stroke, acute myocardial infarct (AMI), and dementia.

**Methods:**

Based on the DMIC, a survey was developed for integrated care coordinators. We invited all Dutch stroke and AMI-services, as well as the dementia care networks to participate, of which 84 did (response rate 83%). Data were collected on relevance, presence, and year of implementation of the 89 elements. The data analysis was done by means of descriptive statistics, Chi Square, ANOVA and Kruskal-Wallis H tests.

**Results:**

The results indicate that the integrated care practice organizations in all three care settings rated the nine clusters and 89 elements of the DMIC as highly relevant. The average number of elements implemented was 50 ± 18, 42 ± 13, and 45 ± 22 for stroke, acute myocardial infarction, and dementia care services, respectively. Although the dementia networks were significantly younger, their numbers of implemented elements were comparable to those of the other services. The analyses of the implementation timelines showed that the older integrated care services had fewer plans for further implementation than the younger ones. Integrated care coordinators stated that the DMIC helped them to assess their integrated care development in practice and supported them in obtaining ideas for expanding their integrated care activities.

**Conclusions:**

Although the patient composites and the characteristics of the 84 participating integrated care services differed considerably, the results confirm that the clusters and the vast majority of DMIC elements are relevant to all three groups. Therefore, the DMIC can serve as a general quality management tool for integrated care. Applying the model in practice can help in steering further implementations as well as the development of new integrated care practices.

## Background

When a patient's needs cannot be covered by one professional or health care provider alone, collaboration between different providers is required. The collaborative efforts and commitment to organize care for a specific patient group in a streamlined way are generally referred to as 'integrated care,' 'coordinated care', 'collaborative care' or 'chronic disease management' programmes. An integrated care service is defined as a coherent and coordinated set of services which are planned, managed and delivered to individual service users across a range of organizations and by a range of co-operating professionals and informal carers [1]. The available range of terminologies for integrated care and for the underlying concept of integration, illustrates the complexity of this topic. Many researchers and policy makers have distinguished many different dimensions of integration, with the most common taxonomies differentiating the type, breadth and degree of integration [2]. For types of integration, the literature differentiates functional integration, organizational integration, professional integration and clinical integration [3-5]. The breath of integration, often defined as 'horizontal, vertical or virtual', refers to the range and type of healthcare services that collaborate to provide the integrated care. For the degree of integration, Leutz [6,7] is the most frequently cited expert and defines the three levels; 'linkage', 'coordination' and 'integration'. The choice of the level of integration depends on the needs and complexity of the client groups, ranging from intense full integration for complex, multi-morbid clients till only linkage of different systems for less complex situations.

The need for integrated care has grown in the past decade. There is an increasing interest in how health care workers, managers and policy makers could implement effective integrated care services. This situation can be explained by multiple developments. Firstly, the increasing numbers of elderly people and those with chronic illnesses require a shift in focus from acute to chronic care. Further, for many diseases the amount of hospital time has declined which raises the need for close and early connections with long term and social care [8,9]. In addition, in multiple countries the majority of the elderly people prefer to live at home as long as possible, which has made well-organized home care, social care, and palliative care more important [10]. Lastly, in a large number of countries the acute, long-term, and social care areas have separate legal and financial systems. This situation often causes fragmentation and an increase in the complexity of the collaboration [10-12]. To summarize, the shifting needs of patients and the way care is organized in a number of countries on both the micro- (patient), meso- (organizational) as macro- (system) level, results in all kinds of fragmentation. The aim of integrated care is therefore to reduce this fragmentation and deliver better results and outcomes of care on multiple dimensions.

### Implementing integrated care

Whereas the rationale for integrated care has been recognized, the implementation of this type of care is often complex. Although much research has been conducted on integrated care, the studies available only address specific settings and patient groups, while their conclusions regarding which elements should be implemented are partially incompatible [13-16]. Systematic reviews and studies of organizational interventions aimed at improving patient care have established that integrated care could improve care processes, patient outcomes and, although more inconclusive, reduce costs [3,17-20]. Glasby [21] describes the importance of implementing integrated care activities on multiple levels. Activities on the operational or individual level are, for example, streamlining information flows and an accurate transfer of patients, while implementation challenges on a tactical or level refer to for instance measuring performance indicators on a care chain level. Further, the commitment of representatives on a strategic level is required for realizing sustainability and (financial) agreements among professionals or organizations. In practice, the project leaders and coordinators of integrated care daily struggle with the question which care elements to implement and in what order. In the past decade a number of quality management models or frameworks like the Chronic Care Model and it's later versions like the Innovative Care for Chronic Conditions Framework and the Expanded Care Model; the Public Health Model, the Continuity of Care model, the Guided Care model, the Kaiser model, the Evercare model, Pfizer approaches, the PACE model, the PRISM model, the Strengths model, the Evaluation Framework for disease management and the European Foundation for Quality Management Model (EFQM) have been developed which could be used by these professionals [2,22-29]. When we select those models that have healthcare specific versions, that are internationally and frequently used and have assumed or proven relations between the models components and better results in health care, only the EFQM quality management model and the Chronic Care Model (CCM) remain. However, these models do not have integrated care as a dominant and generic perspective. The EFQM quality management model primarily concentrates on the dynamics within organizations and not on interorganizational care pathways [29]. And although the CCM may be more helpful, it is aimed at chronic patient groups, leaving integrated care with also acute aspects (such as trauma care) out of scope [14,15]. In a previous study we therefore developed a quality management model for integrated care, called the Development Model for Integrated Care (DMIC) [30,31].

### The Development Model for Integrated Care

The evidence- and expert-based Development Model for Integrated Care consists of 89 elements grouped in nine clusters. The elements represent a wide range of activities considered as relevant to the realization of integrated care. The clusters are named as follows: 'patient-centeredness', 'delivery system', 'performance management', 'quality of care', 'result-focused learning', 'interprofessional teamwork', 'roles and tasks', 'commitment', and 'transparant entrepreneurship' (see additional file 1). Implementing the elements of all nine clusters contributes to the further development of integrated care. The model intends to be generic and suitable for diverse patient groups that make use of both chronic and acute care services. The model has the potential to serve as an assessment tool for health care professionals, managers and integrated care coordinators to support the implementation of improvement activities. In this study we have empirically tested our theoretical expert-based model in three different integrated care contexts in The Netherlands: stroke, acute myocardial infarction, (AMI), and dementia services. Our research question is:

To what extent are the elements of the Development Model for Integrated Care relevant to and implemented in the integrated care practices for stroke, acute myocardial infarction, and dementia patients?

### Introduction to integrated stroke, AMI, and dementia care

In The Netherlands, with its population of 16 million people, every year about 41,000 people suffer from a stroke. In 2005 22% of the people with a stroke died within one year after their hospital admission [32]. A large number of disciplines and health care providers are involved in stroke care, which consists of three phases. In the acute phase general practitioners, ambulances and hospitals (the emergency department and the stroke unit) are involved. In the rehabilitation phase rehabilitation centres, nursing homes and home care organizations are the care providers. While informal care and patient federations are relevant during the whole care continuum, they become even more important in the chronic phase to support the patients and their families. 'Stroke services' have existed in The Netherlands since the late 1990s and are organized as a network of service providers working together in a structured way to provide adequate services in all stages of the follow-up care for stroke patients [33]. During the last ten years there have been multiple projects to stimulate the development of regional stroke services in The Netherlands. Examples are the Breakthrough Collaboratives, the development of a national indicator set and a stroke benchmark, updated stroke guidelines, and the start of the National Stroke Service Network [34,35]. Nevertheless, there is still room for improvement, while bottlenecks are observed in issues such as the exchange of (electronic record) information among professionals, accurate services in the chronic phase, and the absence of integral financial budgets.

Each year, 36,000 patients suffer from AMI in The Netherlands. Here, approximately 25% of the patients die before reaching the hospital [36]. The current standard treatment for AMI patients is primary percutaneous coronary intervention (PCI), which requires a quick transfer of the patient to a hospital with interventional capacities. International guidelines state that the time interval between the first medical contact and the start of the treatment should not be longer than 90-120 minutes [37]. Given that not every hospital is equipped with interventional capacities, close collaboration is necessary to ensure optimal patient flows through the care chain. The different care providers have made agreements on pre-hospital diagnosis, direct transfer to a catheterization laboratory, bypassing general hospitals and emergency departments, and post intervention patient management. Examples of these care providers are ambulance services, cardiac care units, catheterization laboratories in PCI centres, interventional and general cardiologists, and general practitioners. However, most agreements are made on an operational level between only two parties. Further applying the concept of integrated care services to acute cardiology may therefore help create a care system that offers more consensus among the parties, thereby providing a better understanding of the role of each health care provider. The past years, the number of hospitals with PCI capacities and acute care facilities for AMI patients has increased. This development can be considered as a challenge for the existing care systems to incorporate additional parties into the current agreements.

The number of people with dementia is rapidly increasing in The Netherlands. Nowadays there are 230,000 dementia patients, while this number will have increased to 550,000 by 2050 [38]. Dementia care is divided into three sectors: general care, mental health care, and long-term care. During the onset and early stages of dementia care, support is mostly provided by primary care practitioners, spouses, relatives and patient federations. For medical diagnostics general practitioners can refer to a hospital's specialist memory clinic or to mental health services. After the diagnosis, local services determine the specific care packages, such as case management, support groups, housekeeping, personal care, respite care or counseling. When living at home is no longer possible, sheltered housing or elderly people wards in nursing homes are options. The past five years the development of integrated dementia care networks has gained a lot of attention. Initiatives to stimulate the integrated dementia care in The Netherlands are the National Dementia Program, the Dementia Front Runner Program (integrated financial budgets), the widespread establishment of local Alzheimer federations, a national dementia indicator set, and the start of the development of a national care standard for dementia [39]. Nevertheless, there is still much room for improvement in this sector. Examples are the early detection of the disease, support after the diagnosis, the implementation and financing of case management, crisis intervention, coordination, timely referrals, and adequate support for the spouses and families.

## Methods

To assess the relevance and implementation of the elements of integrated care, we constructed a survey study, based on the Development Model for Integrated Care. We had already designed the Development Model for Integrated Care in two previous studies [16,17] by combining a structured literature study, a Delphi study, and a Concept Mapping study. The literature study of integrated care elements resulted in 101 items. Each element represents an activity aimed at the development (realization, improvement, innovation or sustainability) of integrated care. The Pubmed and Cochrane databases were searched for recent reviews, articles, and multiple other sources, such as PhD theses, evaluation reports, while frequently used quality management models were also studied. After the literature study, we conducted a Delphi study. During three rounds, 31 experts on integrated care rated the importance of the 101 elements by using an ordinal scale (range: 1 = not important; 4 = very important). Next, they improved, completed and confined the list of elements. Each included element was rated by at least 80% of the experts as (very) important for integrated care. This systematic approach resulted in 89 elements of integrated care, grouped in nine clusters. For the grouping procedure Concept Mapping was used. The individual clustering of the experts served as input for multidimensional scaling and hierarchical cluster analysis, resulting in a cluster map with nine clusters of 3 to 18 elements.

For the present study we constructed an Excel-based questionnaire. The first part (A) of the questionnaire focused on general information about the integrated care practice, such as the year when the collaboration had started, the number of patients in the year prior to that year, the number and type of health care providers involved, the current agreements among the care providers, infrastructures for cooperation improvement, the availability of a coordinator on the care chain level, and the commitment on a strategic level. The second part of the questionnaire (B) concerned the clusters and elements of the model. The respondents were asked to rate whether each element was relevant to their specific integrated care practice (yes = 1, no = 0) and if so, whether and in which year this element was implemented. The maximum relevance score on a cluster level for the total group was 1, the elements having equal weights. If elements were not implemented, there was an option by which to indicate that there were intentions to implement this element shortly (this year or the next). At the end of section B respondents had the option to add general comments or make suggestions for missing elements. Project leaders or coordinators of integrated stroke services as well as AMI and dementia care networks were invited to fill in the questionnaire. To assure that the right respondents took part, we clearly explained the criteria for participation via personal contact or sometimes by visiting them. The rationale for investigating these three different patient groups was based on multiple criteria. Firstly, we wanted variance among the participating integrated care services to assess the generalizability of the model. This variety had to apply to both the different client groups and their different care providers from the various sectors (acute care, chronic care, and social care). The AMI group has a strong focus on acute care settings, while the stroke group covers the entire continuum from acute to chronic care. The slow and progressive syndrome of dementia also includes mental health care and social care. Next, to include integrated care services in different stages of development, the years had to vary when the integration had been started. This was indeed the case for the three groups: dementia has only more recently received attention in The Netherlands, whereas AMI and stroke services have already been offered for a longer period of time. Another criterion was the inclusion of collaborative national networks that were willing to stimulate participation. The National Stroke Service Network, the National Network on Dementia, and the National Society for Trauma Centers all recommended participation in a letter to their members. Another criterion was geographical spread. This criterion was met since the national networks operate in most parts of the country. Finally, a coordinator on the tactical level was required. In all three sectors this criterion was met by a majority of the integrated care services. We contacted these coordinators and asked for their participation in the study. Each service was asked to fill in one questionnaire. The criteria for the respondents were that they had a good overview of the current state, history, and future plans of the integrated care service as a whole. The respondents had to participate on behalf of all integrated care providers involved and were allowed to contact colleagues in their integrated care setting to help them answer the questions. For this study, no ethical approval was needed. The collected data did not address any individual nor group wise patient data. The focus was on organisational aspects of integrated care (the 89 elements) which were delivered on a voluntary basis by the integrated care coordinators.

Ultimately 36 stroke services, 50 dementia care networks, and 12 myocard services were invited to participate in our study. Upon acceptation of our invitation, the respondents received the Excel-based questionnaire and an instruction sheet by e-mail. Non-responders were reminded twice, by telephone and by e-mail. Due to its smaller scale, the organizations in the AMI service sectors were visited beforehand by one of the researchers to introduce them to the questionnaire. Non-responders to our first call were telephoned by the researchers to explain the purpose of the study, after which they asked again for their participation. If indicated on the questionnaire, the reasons for the non-response as well as additional remarks were documented.

The data analyses were executed per service and for the total group by means of descriptive statistics, frequency analyses, Chi Square, ANOVA and Kruskal-Wallis H, using SPSS software, version 16.0.

## Results

### Participating integrated care services

The overall response rate to the questionnaire was 83%; 32 of the 36 stroke services participated (89%), 9 of the 12 AMI services (75%) and 43 of the 50 dementia services (86%). Reasons for non-response were a lack of time to answer the questionnaire or absense of the service coordinator. Respondents stated that filling in the questionnaire took about 30 to 45 minutes. Table 1 contains the characteristics of the participating integrated care services. The average year when integrated care was first started ranged between 2001 (stroke) and 2007 (dementia). The average number of stroke patients who entered the stroke services in 2008 was 449 ± 340 (range 134-1914). For the AMI group on average 1109 ± 515 patients (range 519-2200) entered the care chain in 2008. For dementia there were no central databases available that indicated the total number of clients per integrated care service. This was because multiple providers can start this care segment. All three services collaborated with hospitals, nursing and elderly homes, home care organizations, and general practitioners in a large number of the cases. Municipalities were involved in a minority of the stroke services (13%), in 72% of the dementia networks, but not in the AMI services. The percentage of services having periodical meetings with the financial bodies involved varied. Meetings with health insurers were held by 19% of the stroke, 11% of the AMI, and 28% of the dementia services. Health insurers are mainly focused on the cure sector, as the long-term care is organized differently in The Netherlands. Insurance companies divide the country into 32 regions, and in each region the largest one acts on behalf of all others as the regional contractor and finance body of the long-term care providers. Regular meetings with these bodies were common for 28% of the stroke, 11% of the AMI, and 93% of the dementia services. Long-term care clients require a needs assessment report from an independent organization before they can receive care from a provider. Twenty five percent of the stroke and 14% of the dementia services had regular contact with these organizations, which did not apply to the AMI services.

**Table 1:** Characteristics of participating integrated care services

Characteristic	Stroke n = 32	AMI n = 9	Dementia n = 43
*Average start year (min - max)*	*2001 (1996-2005)*	*2003 (1998-2004)*	*2007 (2000 - 2010)*

*Average lifespan in years (sd)*	*7.75 ± 2.4*	*5.67 ± .2.0*	*2.72 ± 2.1*

*Involved care providers (% of n):*			
- *hospitals*	*100%*	*100%*	*91%*
- *expertise center*	*---*	*---*	*47%*
- *mental health care*	*0%*	*0%*	*98%*
- *rehabilitation center*	*88%*	*0%*	*0%*
- *nursing and elderly homes*	*100%*	*11%*	*100%*
- *home care*	*100%*	*0%*	*100%*
- *welfare/social care*	*---*	*0%*	*77%*
- *client organisation* - *municipality*	*38%* *13%*	*0%* *0%*	*98%* *72%*

*Agreements available with: (% of n)*			
- *general practitioners*	*72%*	*89%*	*56%*
- *ambulances*	*78%*	*100%*	*0%*

*% with integrated care coördinator*	*78%*	*33%*	*96%*
*Average hours per week (min-max)*	*5.5 (0-19)*	*2.0 (1.5-2.4)*	*15.0 (2-36)*

*% with improvement teams on care*	*91%*	*78%*	*91%*
*chain level, consisting of*	*3%*	*100%*	*13%*
*- professionals*	*3%*	*0%*	*3%*
*- managers*	*93%*	*0%*	*85%*
*- mixed*			

*% with formal collaboration agreement between involved providers*	*69%*	*22%*	*84%*

*% with regular board meetings of involved providers*	*78%*	*67%*	*95%*

### Relevance of the elements

**Figure 1:**
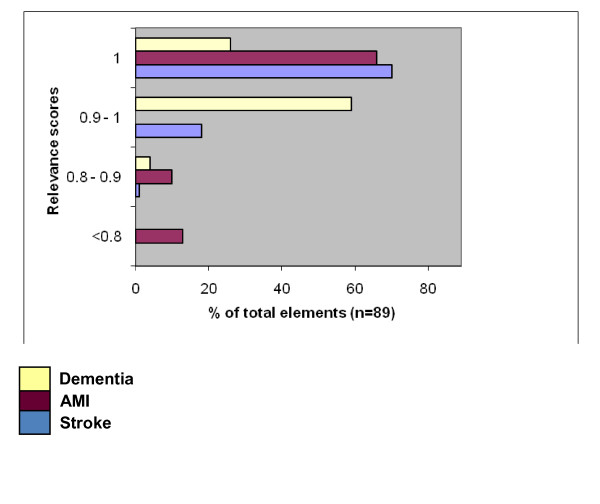
**Relevance scores of elements**.

For all 89 elements relevance scores (RS) were calculated. Overall, the relevance of the elements was high in the case of all three integrated care settings. As regards stroke and dementia, all elements could be classified as relevant at a cut-off point of 80%, as in our previous Delphi study (see Figure 1). For the AMI services 13 elements scored lower than 80%. Six of these were assessed as relevant by 78% of the respondents. Four elements scored lower than 50%, namely 'developing care programmes for relevant client subgroups'(44%); 'developing criteria for assessing clients' urgency' (33%); 'reaching agreements among care partners on scheduling client examinations and treatment' (22%) and 'reaching agreements among care partners on providing care to waiting-list clients' (11%). For the total group the relevance scores on a cluster level were between 0.9 and 1, which meant high relevance scores for all clusters. For the three subgroups, the scores ranged between 0.98 and 1.0 (stroke); 0.78 and 1.0 (AMI) and 0.95 and 0.99 (dementia), see also table 2. Three of the respondents named a missing element after finishing the questionnaire, but the elements were very close related to those already in the set.

**Table 2:** Relevance scores per cluster

Cluster (nr of elements)	Total	Stroke	AMI	Dementia
***1. Client centeredness (9)***	0.93	0.98	0.83	0.98
1		7	3	5
0.9 - 1		1	0	4
0.8 - 0.89		1	2	0
< 0.8		0	4	0

*2. Delivery system (18)*	0.90	0.98	0.78	0.95
1		12	8	5
0.9 - 1		6	0	11
0.8 - 0.89		0	3	2
< 0.8		0	7	0

*3. Performance management (16)*	0.98	0.99	1.0	0.95
1		13	16	0
0.9 - 1		3	0	14
0.8 - 0.89		0	0	3
< 0.8		0	0	0

*4. Quality care (5)*	0.95	0.99	0.91	0.96
*1*		4	2	1
0.9 - 1		1	0	4
0.8 - 0.89		0	2	0
< 0.8		0	1	0

*5. Result-focused learning (12)*	0.99	0.99	1.0	0.97
1		12	12	1
0.9 - 1		8	0	11
0.8 - 0.89		0	0	0
< 0.8		0	0	0

*6. Interprofessional teamwork (3)*	0.99	0.99	1.0	0.98
1		2	3	1
0.9 - 1		1	0	2
0.8 - 0.89		0	0	0
< 0.8		0	0	0

*7. Roles and tasks (8)*	0.99	1.0	0.99	0.97
1		8	7	0
0.9 - 1		0	0	8
0.8 - 0.89		0	1	0
< 0.8		0	0	0

*8. Commitment (11)*	0.99	0.99	0.99	0.99
1		9	10	8
0.9 - 1		2	0	3
0.8 - 0.89		0	1	0
< 0.8		0	0	0

*9. Transparant entrepreneurship (7)*	0.98	1.0	0.95	0.99
1		7	5	5
0.9 - 1		0	0	2
0.8 - 0.89		0	1	0
< 0.8		0	1	0

### Implementation of the elements

**Figure 2:**
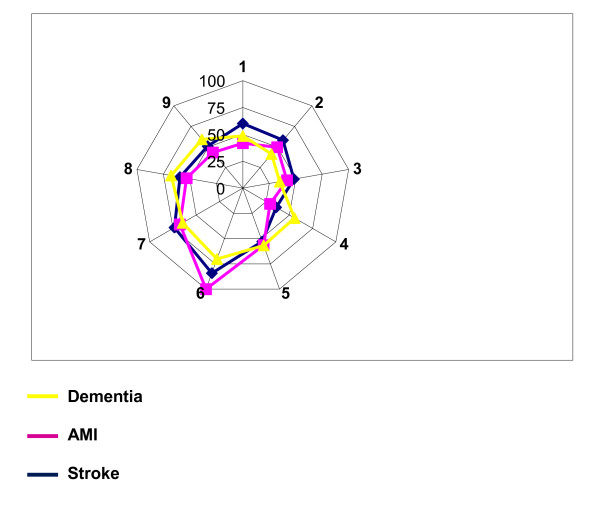
**Percentage of relevant implemented elements per cluster**. Cluster names: 1 = patient-centeredness. 2 = delivery system. 3 = performance management. 4 = quality of care. 5 = result-focused learning. 6 = interprofessional teamwork. 7 = roles and tasks. 8 = commitment. 9 = transparant entrepreneurship.

The number of implemented elements of the Development model for Integrated Care varied within and among the three services. The average number of elements (maximum 89) for the total group was 46 ± 20 items (range 3-82). For the three subgroups, the amounts ranged from 50 ± 18 (10-77) elements for stroke, 42 ± 13 (20-61) elements for AMI, and 45 ± 22 (3-82) for dementia. Figure 2 gives an overview of the percentages of the implemented elements per cluster that were rated as relevant by the respondents. For the total group, the mean percentages of these elements were the highest in the 'inter-professional teamwork' (85 ± 29) and in the 'roles and tasks' clusters (69 ± 29). The implemented elements with the lowest relevance percentages were found in the clusters 'quality care' (40 ± 24) and 'performance management' (42 ± 30). The mean numbers of the elements marked as 'planned for the near future' differed significantly among the stroke, AMI, and dementia services (respectively 8, 4 and 21, p < 0.001). When we look at the timespan of the implemented elements, the dementia services show the most recent dates, with most elements implemented between 2007 and 2009. For both stroke and AMI most elements were implemented between 2002 and 2006. Analyses of the correlation between the relevance scores of elements and the implemented elements showed no correlation (r = -0.02, p ≥ 0,10). Additional file 1 presents the implementation scores, the average year of implementation, and the percentages of the plans for working on the elements.

## Discussion

The results of this study indicate that the integrated care practice widely recognizes the Development Model for Integrated Care with its evidence- and expert-based elements and clusters. Regardless of the differences among the three integrated care services (stroke, AMI, and dementia patients) who differed in age, client groups, size, focus on either acute or chronic care, collaboration infrastructure, and the care providers involved, they all rated the elements of the Development model for Integrated Care as highly relevant. Based on these results we may conclude that the empirical test of our theoretical model has been successful and that this tool has the potential to effectively support multiple integrated care practices.

In addition to the useful information gathered regarding the relevance and implementation of the elements of integrated care, a large number of respondents gave feedback on the model's applicability. The integrated care coordinators indicated that filling in the questionnaire was a good exercise to reflect upon the current situation. Discussing the implementation of the elements gave new ideas for the improvement and further development of their integrated care practice. The respondents used the elements and clusters for their quality management systems, improvement plans or even wrote a discussion paper for their steering committee based on the questionnaire results.

Although the relevance scores were all (very) high, some important differences were observed among the nine clusters. For the AMI services three elements of cluster 2 ('delivery system') had the lowest relevance scores (< 50%). The average priority score of these four elements was 1.94, which is markedly lower than the average of 2.23 of the whole set (see also additional file 1). When analyzing the content of these elements, however, it made sense that 'providing care to waiting list patients' and 'criteria for urgency' do not apply to this client group, since these items are associated with the provision of acute care. 'Providing case management', another low scoring element in the case of AMI services, generally applies to clients who need multidisciplinary care during a prolonged period of time. Case management is one of the crucial interventions currently implemented in The Netherlands for dementia patients [40]. This situation corresponds with our study findings; a large number of services have already implemented case management or are planning on introducing this approach.

It can be concluded that integrated care settings are generally still in a developmental stage. Especially in the dementia services, the number of planned elements is high. On average half of the elements identified have been implemented in practice. And within all three service groups the integrated care services vary in their plans and implementation rates. The absence of correlations between the relevance scores of implemented elements and their implementation rates could be explained by the overall high scores with little variation between relevant scores. It assumes that choices for interventions are influenced by other factors like possibly the amount of development or 'maturity' of the integrated care service. In an earlier, more conceptual study [31] we concluded that integrated care services can experience different phases of development which are called: 'the initiative and design phase', 'the experimental and execution phase', 'the expansion and monitoring phase' and 'the consolidation and transformation phase'. It would be interesting to further research a possible relation between the phases of development and the implementation rates. The AMI services are strongly focused on the professional and more practical level, as illustrated by high scores in the clusters 'inter-professional teamwork' and 'roles and tasks', which refer to the earlier phases of development. Since the AMI-services have not included the rehabilitation phase after an infarction, the next step may be the expansion to a full service. In this case, the AMI services would also be faced with some of the bottlenecks typical of the later phases of development, such as separate financial systems and the need for formal agreements among providers. However, AMI has not yet made many plans in this direction. This situation may be explained by the absence of a coordinator in the majority of the AMI organizations.

The stroke service provides a broad spectrum of integrated care consisting of a substantial number of integrated care elements. Although stroke represents one of the first and 'oldest' patient groups for which integrated care was developed on a large scale, some of its activities still seem to be in their initial stage. Elements from the clusters 'performance management' and 'quality care' have not been implemented on a large scale yet. Especially the elements associated with monitoring the quality and results of the care chain and the involvement of clients in assessing their needs and judgements have not yet received sufficient attention. In addition, incentives on a governmental level to further develop these activities are lacking, as there are still no financial or professional stimuli included in the policies for the integrated stroke care in The Netherlands. Despite this fact, the post-stroke mortality rate declined by 25% during the period 2000 - 2005, which is believed to be a result of the introduction of stroke services and more precise diagnostics and treatment approaches [41].

Dementia services were initiated significantly later than stroke and AMI, but the number of elements already implemented indicates that this segment has developed rapidly during the past years. This process is accompanied by a focus on integrated budgets, experiments, and formal agreements (as indicated in cluster 9). We assume that national initiatives, such as the National Dementia Program, the Front Runner Program, and a strong nationwide network of client federations have accelerated the development of this service. In addition, there are many plans for the near future, which has raised the expectation for the coming years. The newness of the concept of integrated care to the people working in the dementia sector may stimulate their enthusiasm in making plans to further develop the service. In other words, the biggest growth of the system possibly lies in the beginning of it.

### Study limitations

Our study has some limitations. First, the number of research participants per patient group differed, which was due to the nature of the current situation. The AMI services were only represented by nine of the twelve services because the number of hospitals with interventional capacities was limited, which means that there were only a few networks. The treatment of a stroke, however, can be initiated in almost any hospital. Second, although a number of respondents consulted partners in the care chain when filling in the questionnaire, it would be interesting to invite more care workers in the three integrated care service to add additional perspectives. Third, the respondents' answers were based on self-reported data. Whenever elements were implemented, these decisions were based on the judgement of the integrated care service representatives themselves without consulting other sources, such as documentation or interviews. Finally, we focused our research on integrated care services in the Netherlands, while it would also be interesting to expand this study internationally.

### Recommendations for research and practice

We have multiple suggestions for further research to further assess the generalizability of the model. Firstly, we suggest to broaden the assessment of the implemented elements by involving multiple professionals, managers and also client representatives per integrated care setting. Adding these perspectives can provide interesting information about how the implementation is being experienced and if consensus is available. Secondly, we suggest repeating the study in integrated care services which focus on other client groups like for instance clients with diabetes, COPD, depression or other groups like frail elderly who need support on broader life domains. This kind of research could provide knowledge about the further applicability of the model, because of our aim was to develop a generic model. A third option could be expanding our research to other countries. Next to changes on the 'meso- or organisational level' of integrated care where our research focuses on, the 'also macro- or system level' characteristics and differences are being taken into account. These characteristics address for instance other political, demographical, legal and professional or educational contexts.

Another suggestion is further research on the different phases of development of integrated care services and the implemented elements in each phase. Previous research revealed different phases of development, but the relation between these phases and the implementation of elements in each phase is less clear. Also, the implementation process of the elements asks for different roles, needed expertises and strategies of integrated care coordinators, professionals and managers. These are interesting topics for further research.

Finally, we suggest follow-up research on the relation between the implementation of the elements and clusters of the DMIC and the delivered results. Do more 'mature' integrated care practices or practices that implemented more elements achieve better results in quality of care, quality of life, client related indicators (or client experiences) and costs?

Our study has a number of practical implications. Coordinators and managers may use the Development model for Integrated Care as a quality management tool in their integrated care practices. The model with its elements and clusters is suitable for different patients groups and can be used as an assessment instrument to monitor the integrated care activities. Moreover, the respondents indicated that the model also worked as a self-evaluation tool and helped them in the formulation of improvement plans. Further use in practice could be enhanced by developing a DMIC-based user-friendly (web based) tool, in which not only integrated care coordinators but also multiple partners working in integrated care services could score the elements on relevance and implementation. By presenting the (consensus) results found, clusters and elements with lower scores could be further discussed and prioritised as a basis for an improvement plan. Managers can use the model in broadening their vision on integrated care and improving their quality management. Furthermore, the model can be used for benchmarking by comparing the (absolute) implementation scores between integrated care practices. Practices can mirror their own results with comparable others and get input for improvement activities. The National Stroke Service network has plans to use the model for auditing its stroke services in The Netherlands in the coming years.

## Conclusion

This study has assessed the practical relevance and implementation of the Development Model for Integrated Care, consisting of nine clusters with in total 89 elements, in three integrated care settings: AMI, stoke, and dementia. These segments varied considerably. The AMI services can be characterized as acute care, while stroke services range from acute to chronic care. Finally, the dementia services merely focus on chronic care. In all three integrated care settings the relevance of the elements was considered high. We can therefore conclude that the Development Model for Integrated Care has a generic character and can serve as a useful tool for assessment, evaluation or improvement in both the research on integrated care and its development in the practical field.

In addition, the study has provided a detailed analysis to what extent integrated care has been implemented within each service and on which topics. The average number of implemented elements was 50 ± 18, 42 ± 13, and 45 ± 22 for stroke, acute myocardial infarction, and dementia care services, respectively. Although the dementia services were significantly newer, the number of implemented elements was comparable to that of the other segments. The average number of planned elements told us that the integrated care services are still developing, although the intensity differs significantly among the three groups. With respect to new initiatives and plans the dementia services take the lead, which might be explained by the national initiatives and incentives in this area and the actions of client federations. Research to further assess the generalizability of the model for other (international) client groups and the relation between integrated care development and the DMIC elements is suggested.

## Competing interests

The authors declare that they have no competing interests.

## Authors' contributions

All authors contributed to the study. MM, KA and RH designed the questionnaire on the basis of previous research. MM and RV collected and analyzed the data, supervised by KA and RH. MM led the writing process, while all authors commented on the sequential drafts before approving the final version of the manuscript.

## Pre-publication history

The pre-publication history for this paper can be accessed here:

http://www.biomedcentral.com/1472-6963/11/177/prepub

## Supplementary Material

Additional file 1 **Implementation of integrated care elements**. This file presents the percentages of implemented elements (PI), the average year of implementation (Ayr), and the percentage of planned elements (PP) of services which have not implemented the element yet. The data were gathered from 32 stroke service (Str), nine AMI-services, and 43 dementia services. PI is based on attainable elements: attainable elements are all services minus those which rated the element as not relevant (see also table 2 for relevance scores). The elements per cluster were ranked by priority scores (PS). These were systematically assessed by an expert panel as described in Minkman et al. 2009 [16]. Maximum priority score is 3.Md = missing data. Click here for file
