# Supplementary material for: The implementation of integrated care: the empirical validation of the Development Model for Integrated Care
Source: BMC Health Serv Res. 2011 Jul 30;11:177. doi: 10.1186/1472-6963-11-177 (PMC3160357; doi:10.1186/1472-6963-11-177)
Supplement: Additional file 1 — Implementation of integrated care elements. This file presents the percentages of implemented elements (PI), the average year of implementation (Ayr), and the percentage of planned elements (PP) of services which have not implemented the element yet. The data were gathered from 32 stroke service (Str), nine AMI-services, and 43 dementia services. PI is based on attainable elements: attainable elements are all services minus those which rated the element as not relevant (see also table 2 for relevance scores). The elements per cluster were ranked by priority scores (PS). These were systematically assessed by an expert panel as described in Minkman et al. 2009 [16]. Maximum priority score is 3.Md = missing data. [file 1472-6963-11-177-S1.DOC]

# Additional file1 – Implementation of integrated care elements

This file presents the percentages of implemented elements (PI), the average year of implementation (Ayr), and the percentage of planned elements (PP) of services which have not implemented the element yet. The data were gathered from 32 stroke service (Str), nine AMI-services, and 43 dementia services. PI is based on attainable elements: attainable elements are all services minus those which rated the element as not relevant (see also table 2 for relevance scores).

The elements per cluster were ranked by priority scores (PS). These were systematically assessed by an expert panel as described in Minkman et al. 2009 [16]. Maximum priority score is 3.

Md=missing data

Cluster 1. Patient-centeredness, 9 elements

| **PS** | **Str**  **PI** | **Str**  **PP** | **Str**  **Ayr** | **Myo**  **PI** | **Myo**  **PP** | **Myo**  **Ayr** | **Dem**  **PI** | **Dem**  **PP** | **Dem**  **Ayr** | **Element description** |
| --- | --- | --- | --- | --- | --- | --- | --- | --- | --- | --- |
| 2.66 | 88 | 75 | 2004 | 71 | 0 | 2005 | 77 | 70 | 2009 | Providing understandable and client-centered information |
| 2.36 | 61 | 25 | 2004 | 0 | 11 |  | 52 | 75 | 2008 | Collaboratively offering client information of the care partners |
| 2.35 | 69 | 10 | 2004 | 63 | 0 | 2005 | 60 | 24 | 2008 | Designing care for clients with multi- or co-morbidities |
| 2.31 | 44 | 28 | 2005 | 33 | 0 | 2007 | 40 | 38 | 2007 | Using self-management support methods as a part of integrated care |
| 2.23 | 56 | 36 | 2002 | 50 | 0 | 2005 | 26 | 66 | 2008 | Implementing care process-supporting clinical information systems |
| 2.13 | 81 | 0 | 2003 | 57 | 0 | 2005 | 61 | 10 | 2008 | Flexible adjustment of integrated care corresponding to individual clients’ needs |
| 2.10 | 36 | 33 | 2004 | 0 | 0 |  | 48 | 67 | 2008 | Developing a front office: single entry point for client information |
| 1.97 | 63 | 67 | 2004 | 67 | 0 | 2002 | 48 | 77 | 2008 | Using a protocol for the systematic follow-up of clients |
| 1.94 | 41 | 21 | 2005 | 50 | 0 | 2006 | 26 | 52 | 2007 | Developing care programmes for relevant client subgroups |
| **Cluster description** | | | | | | | | | | This cluster is aimed at developing integrated care and information flows tailored to specific (sub)groups of patients. The elements focus on providing information on integrated patient and care process support (front offices, self-management support or information systems), and on delivering care adjusted to individual needs (e.g. multi-morbidity). |

Cluster 2. Delivery system, 18 elements

| **PS** | **Str**  **PI** | **Str**  **PP** | **Str**  **Ayr** | **Myo**  **PI** | **Myo**  **PP** | **Myo**  **Ayr** | **Dem**  **PI** | **Dem**  **PP** | **Dem**  **Ayr** | **Element description** |
| --- | --- | --- | --- | --- | --- | --- | --- | --- | --- | --- |
| 2.94 | 97 | 100 | 2002 | 100 | 0 | 2004 | 65 | 93 | 2008 | Reaching agreements on referrals and the transfer of clients through the care chain |
| 2.84 | 88 | 50 | 2003 | 56 | 0 | 2004 | 43 | 96 | 2008 | Reaching agreements on procedures for information exchange |
| 2.71 | 41 | 42 | 2005 | 0 | 29 |  | 13 | 54 | 2009 | Using a single client-monitoring record accessible to all care partners |
| 2.46 | 77 | 57 | 2001 | 78 | 0 | 2004 | 51 | 90 | 2008 | Reaching agreements on procedures for the exchange of client information |
| 2.42 | 13 | 31 | 2008 | 11 | 38 | 2008 | 5 | 46 | 2010 | Developing connections with the databases of partners in the care chain |
| 2.38 | 22 | 8 | 2003 | 0 | 0 |  | 81 | 88 | 2009 | Offering case management to clients with complex needs |
| 2.32 | 88 | 75 | 2003 | 78 | 0 | 2003 | 38 | 58 | 2007 | Reaching agreements on chain logistics (e.g. waiting periods and throughput times) |
| 2.32 | 19 | 12 | 2005 | 20 | 0 | 2008 | 30 | 61 | 2009 | Using shared client treatment and care plans |
| 2.26 | 31 | 25 | 2004 | 13 | 29 | 1990 | 13 | 43 | 2008 | Using uniform client-identification numbers within the care chain |
| 2.19 | 59 | 38 | 2003 | 89 | 0 | 2003 | 56 | 89 | 2007 | Reaching agreements among care partners on the consultation of experts and professionals |
| 2.07 | 66 | 0 | 2003 | 57 | 0 | 2004 | 41 | 52 | 2007 | Reaching agreements among care partners on managing client preferences |
| 2.06 | 91 | 0 | 2001 | 50 | 0 | 1998 | 53 | 50 | 2009 | Reaching agreements among care partners on scheduling client examinations and treatment |
| 2.05 | 81 | 0 | 2002 | 78 | 0 | 2003 | 32 | 38 | 2008 | Reaching agreements among care partners on discharge planning |
| 2.00 | 94 | 100 | 2002 | 88 | 0 | 2002 | 57 | 44 | 2008 | Developing criteria for the inclusion and throughput of clients in the care chain |
| 1.97 | 28 | 0 | 2003 | 0 | 0 |  | 38 | 57 | 2007 | Reaching agreements among care partners on providing care to waiting-list clients |
| 1.95 | 39 | 16 | 2002 | 0 | 0 |  | 54 | 42 | 2007 | Deploying specialized nurses within the care chain |
| 1.94 | 63 | 25 | 2002 | 38 | 0 | 2005 | 41 | 43 | 2008 | Reaching agreements on linking clients to outside resources or community care partners |
| 1.79 | 47 | 6 | 2002 | 67 | 0 | 1999 | 27 | 47 | 2005 | Developing criteria for assessing clients’ urgency |
| **Cluster description** | | | | | | | | | | Chain and client logistics, coordination mechanisms and procedures for streamlining the care process for the whole care chain is the main focus of this cluster. Further objectives are reaching consensus regarding all agreements (e.g. logistics, sharing expertise), procedures (e.g. information exchange) or tools (e.g. care plans) required from the client’s initial entry into the care chain until the final phase. |

Cluster 3. Performance management, 16 elements

| **PS** | **Str**  **PI** | **Str**  **PP** | **Str**  **Ayr** | **Myo**  **PI** | **Myo**  **PP** | **Myo**  **Ayr** | **Dem**  **PI** | **Dem**  **PP** | **Dem**  **Ayr** | **Element description** |
| --- | --- | --- | --- | --- | --- | --- | --- | --- | --- | --- |
| 2.55 | 84 | 60 | 2003 | 67 | 33 | 2005 | 45 | 65 | 2009 | Defining performance indicators to evaluate the results of the integrated care delivered |
| 2.50 | 72 | 11 | 2004 | 78 | 50 | 2004 | 44 | 65 | 2007 | Providing feedback to care partners on transfers |
| 2.44 | 47 | 35 | 2004 | 44 | 60 | 2000 | 28 | 45 | 2007 | Gathering client-related performance data (health status, quality of life) |
| 2.42 | 88 | 75 | 2004 | 67 | 33 | 2002 | 50 | 76 | 2009 | Gathering data on client logistics (e.g. volumes, waiting periods and throughput times) in the care chain |
| 2.41 | 47 | 6 | 2004 | 56 | 0 | 2005 | 39 | 44 | 2007 | Using feedback and reminders by professionals for improving care |
| 2.40 | 50 | 25 | 2006 | 33 | 50 | 2007 | 18 | 64 | 2009 | Reaching agreements about the uniform use of performance indicators in the care chain |
| 2.39 | 47 | 35 | 2003 | 33 | 17 | 2004 | 60 | 65 | 2009 | Monitoring successes and results during the development of the integrated care chain |
| 2.33 | 48 | 38 | 2004 | 22 | 14 | 2004 | 17 | 66 | 2009 | Establishing quality targets for the performance of the whole care chain |
| 2.32 | 3 | 10 | md | 33 | 0 | 2006 | 13 | 24 | 2007 | Monitoring and analyzing mistakes/near mistakes in the care chain |
| 2.27 | 39 | 30 | 2004 | 11 | 25 | 2008 | 38 | 58 | 2009 | Using a systematic procedure for the evaluation of agreements, approaches and results |
| 2.25 | 16 | 33 | 2005 | 0 | 11 |  | 38 | 77 | 2009 | Monitoring client judgements and satisfaction for the whole care chain |
| 2.23 | 13 | 18 | 2006 | 11 | 0 | 2004 | 31 | 66 | 2009 | Gathering financial performance data of the care chain |
| 2.19 | 31 | 9 | 2004 | 11 | 13 | 2008 | 17 | 35 | 2009 | Making transparent the effects of the collaboration on the production of the care partners |
| 2.19 | 56 | 36 | 2004 | 89 | 0 | 2004 | 27 | 48 | 2006 | Monitoring whether the care delivered corresponds with the evidence-based guidelines |
| 2.18 | 50 | 31 | 2003 | 33 | 50 | 2004 | 17 | 51 | 2009 | Establishing quality targets for the performance of care partners |
| 1.98 | 81 | 17 | 2004 | 78 | 0 | 2007 | 74 | 55 | 2009 | Installing improvement teams at the care-chain level |
| **Cluster description** | | | | | | | | | | Measurement and analyses of the results of the care delivered in the care chain is the central theme of this cluster. The elements address performance targets at all levels, monitored by the standardized use of indicators. The indicators refer to client outcomes, client judgments, organizational outcomes, and financial performance data. (Near) mistake analysis, feedback mechanisms and improvement teams are used to improve and manage the level of performance |

Cluster 4. Quality care, 5 elements

| **PS** | **Str**  **PI** | **Str**  **PP** | **Str**  **Ayr** | **Myo**  **PI** | **Myo**  **PP** | **Myo**  **Ayr** | **Dem**  **PI** | **Dem**  **PP** | **Dem**  **Ayr** | **Element description** |
| --- | --- | --- | --- | --- | --- | --- | --- | --- | --- | --- |
| 2.65 | 13 | 11 | 2004 | 0 | 14 |  | 46 | 45 | 2008 | Systematically assessing the needs of the clients in the care chain |
| 2.55 | 44 | 33 | 2004 | 33 | 17 | 2004 | 24 | 55 | 2007 | Developing a multidisciplinary care pathway |
| 2.43 | 28 | 30 | 2005 | 0 | 13 |  | 81 | 63 | 2009 | Involving client representatives in improvement projects in the care chain |
| 2.40 | 66 | 45 | 2004 | 100 | 0 | 2004 | 50 | 60 | 2007 | Using evidence-based guidelines and standards |
| 2.12 | 28 | 22 | 2005 | 0 | 13 |  | 74 | 64 | 2008 | Involving client representatives in monitoring the performance of the care chain |
| **Cluster description** | | | | | | | | | | This cluster contains elements that focus on the design of a multidisciplinary care pathway throughout the care chain, based on evidence-based guidelines and standards, as well as clients’ needs and preferences. For this purpose a needs assessment of the specific client group is required, combined with the involvement of client representatives in designing, improving, and monitoring the integrated care. |

Cluster 5. Result-focused learning, 12 elements

| **PS** | **Str**  **PI** | **Str**  **PP** | **Str**  **Ayr** | **Myo**  **PI** | **Myo**  **PP** | **Myo**  **Ayr** | **Dem**  **PI** | **Dem**  **PP** | **Dem**  **Ayr** | **Element description** |
| --- | --- | --- | --- | --- | --- | --- | --- | --- | --- | --- |
| 2.37 | 68 | 30 | 2003 | 67 | 33 | 2006 | 79 | 44 | 2007 | Stimulating a learning culture and continuous improvement in the care chain |
| 2.27 | 56 | 0 | 2004 | 78 | 0 | 2005 | 62 | 0 | 2009 | Defining and assessing the characteristics of the collaboratively delivered care |
| 2.26 | 38 | 15 | 2003 | 22 | 0 | 2008 | 38 | 38 | 2008 | Making the benefits of the collaboration transparent for each care-chain partner |
| 2.23 | 84 | 40 | 2004 | 78 | 0 | 2007 | 79 | 89 | 2008 | Collaboratively assessing bottlenecks and gaps in care |
| 2.17 | 56 | 29 | 2004 | 89 | 0 | 2006 | 62 | 75 | 2008 | Sharing knowledge among care partners about effectively organizing sustainable integrated care |
| 2.16 | 74 | 13 | 2003 | 67 | 33 | 2007 | 80 | 44 | 2007 | Striving toward an open culture for discussing possible improvements for care partners |
| 2.14 | 61 | 8 | 2004 | 67 | 33 | 2006 | 76 | 50 | 2008 | Learning by the exchange of information among professionals about the care process |
| 2.13 | 3 | 6 | 2000 | 11 | 0 | 2008 | 12 | 33 | 2008 | Integrating incentives for rewarding the achievement of quality targets |
| 2.11 | 44 | 0 | 2002 | 78 | 0 | 2004 | 57 | 44 | 2008 | Using knowledge and information for directing and coordinating the care chain |
| 2.11 | 69 | 50 | 2003 | 44 | 40 | 2004 | 52 | 35 | 2008 | Introducing collaborative education programmes and learning environments for the care professionals |
| 2.03 | 13 | 14 | 2002 | 22 | 14 | 2004 | 17 | 38 | 2008 | Linking consequences to the achievement of goals agreed upon |
| 1.88 | 71 | 11 | 2003 | 56 | 25 | 2006 | 64 | 47 | 2009 | Collaborative learning in the care chain in order to innovate integrated care |
| **Cluster description** | | | | | | | | | | The central theme of this cluster is establishing a learning climate aimed at continuously  improving the results in the care chain The elements address essential ingredients for improvement:  defining goals for collaboration, identifying bottlenecks and gaps in care, and ways of learning  and exchanging knowledge in an open atmosphere. Incentives are used to reward improved  performance. |

Cluster 6. Interprofessional teamwork, 3 elements

| **PS** | **Str**  **PI** | **Str**  **PP** | **Str**  **Ayr** | **Myo**  **PI** | **Myo**  **PP** | **Myo**  **Ayr** | **Dem**  **PI** | **Dem**  **PP** | **Dem**  **Ayr** | **Element description** |
| --- | --- | --- | --- | --- | --- | --- | --- | --- | --- | --- |
| 2.61 | 90 | 33 | 2003 | 100 | 0 | 2004 | 86 | 50 | 2007 | Defining the targeted client group |
| 2.26 | 100 | 0 | 2001 | 100 | 0 | 2004 | 67 | 57 | 2008 | Working in multidisciplinary teams |
| 2.04 | 63 | 25 | 2003 | 100 | 0 | 2004 | 59 | 65 | 2007 | Reaching agreements on the availability and accessibility of professionals |
| **Cluster description** | | | | | | | | | | This cluster represents interprofessional teamwork for a well-defined client group. This client group forms the target of the collaborating professionals, who work in well-organized multidisciplinary teams in the care chain. |

Cluster 7. Roles and tasks, 8 elements

| **PS** | **Str**  **PI** | **Str**  **PP** | **Str**  **Ayr** | **Myo**  **PI** | **Myo**  **PP** | **Myo**  **Ayr** | **Dem**  **PI** | **Dem**  **PP** | **Dem**  **Ayr** | **Element description** |
| --- | --- | --- | --- | --- | --- | --- | --- | --- | --- | --- |
| 2.55 | 78 | 29 | 2003 | 67 | 33 | 2006 | 69 | 69 | 2008 | Reaching agreements among care partners on tasks, responsibilities and authorizations |
| 2.55 | 91 | 33 | 2002 | 89 | 0 | 2006 | 76 | 80 | 2009 | Achieving adjustments among care partners by means of direct contact |
| 2.36 | 84 | 0 | 2003 | 89 | 0 | 2007 | 64 | 87 | 2009 | Ensuring that professionals in the care chain are informed of one another’s expertise and tasks |
| 2.20 | 78 | 29 | 2005 | 56 | 25 | 2006 | 83 | 71 | 2008 | Installing a coordinator working at the chain-care level |
| 2.18 | 75 | 13 | 2002 | 78 | 0 | 2006 | 52 | 50 | 2008 | Establishing the roles and tasks of multidisciplinary team members |
| 2.13 | 69 | 20 | 2004 | 78 | 0 | 2006 | 71 | 67 | 2008 | Realizing direct contact among professionals in the care chain |
| 2.07 | 34 | 33 | 2003 | 25 | 0 | 2004 | 24 | 59 | 2007 | Reaching agreements on introducing and integrating new partners in the care chain |
| 2.05 | 78 | 29 | 2003 | 56 | 0 | 2007 | 80 | 50 | 2008 | Directing the care chain by appointing a limited number of people with coordinating tasks |
| **Cluster description** | | | | | | | | | | This cluster reflects the need for clarity about one another’s expertise, roles and tasks in the care chain. The main target issues are an effective collaboration at all levels with (new) partners and a proper allocation of the coordinating tasks. |

Cluster 8. Commitment, 11 elements

| **PS** | **Str**  **PI** | **Str**  **PP** | **Str**  **Ayr** | **Myo**  **PI** | **Myo**  **PP** | **Myo**  **Ayr** | **Dem**  **PI** | **Dem**  **PP** | **Dem**  **Ayr** | **Element description** |
| --- | --- | --- | --- | --- | --- | --- | --- | --- | --- | --- |
| 2.49 | 81 | 50 | 2004 | 67 | 0 | 2006 | 81 | 88 | 2008 | Defining the ambitions and aims of the collaboration in the care chain |
| 2.43 | 72 | 44 | 2003 | 44 | 40 | 2006 | 69 | 92 | 2009 | Signing collaboration agreements among the care partners |
| 2.40 | 84 | 80 | 2003 | 67 | 0 | 2007 | 71 | 83 | 2008 | Assuring the leadership commitment of the partners involved in the care chain |
| 2.29 | 38 | 30 | 2006 | 22 | 0 | 2007 | 58 | 50 | 2009 | Describing the tasks and authorities of leaders, coordinators and advisory boards in the care chain |
| 2.19 | 50 | 25 | 2003 | 56 | 25 | 2006 | 48 | 38 | 2009 | Establishing dependencies among care partners |
| 2.17 | 72 | 44 | 2003 | 56 | 0 | 2006 | 72 | 67 | 2009 | Guiding the care chain by emphasizing a collaborative commitment |
| 2.16 | 75 | 25 | 2003 | 67 | 0 | 2008 | 88 | 40 | 2008 | Structural meetings of the leaders of the care-chain organizations |
| 2.08 | 22 | 16 | 2005 | 56 | 25 | 2006 | 42 | 48 | 2008 | Reaching agreements about letting go care partner domains |
| 2.07 | 56 | 36 | 2005 | 56 | 0 | 2006 | 67 | 36 | 2007 | Stimulating trust among care partners |
| 2.04 | 90 | 33 | 2004 | 67 | 33 | 2006 | 70 | 69 | 2009 | Stimulating the awareness of working in a care chain |
| 1.91 | 13 | 15 | 2005 | 25 | 0 | 2007 | 77 | 50 | 2008 | Structural meetings with external parties, such as insurers, local governments and inspectorates |
| **Cluster description** | | | | | | | | | | This cluster’s focus is on collaborative commitment and ambition in the care chain. In addition to the awareness of dependencies and domains, other target items are commitment toward clearly defined goals and a collaboration ambition. Other components are the general awareness of working in a care chain and the commitment of leaders in this trajectory. |

Cluster 9. Transparent entrepreneurship, 7 elements

| **PS** | **Str**  **PI** | **Str**  **PP** | **Str**  **Ayr** | **Myo**  **PI** | **Myo**  **PP** | **Myo**  **Ayr** | **Dem**  **PI** | **Dem**  **PP** | **Dem**  **Ayr** | **Element description** |
| --- | --- | --- | --- | --- | --- | --- | --- | --- | --- | --- |
| 2.59 | 59 | 23 | 2004 | 44 | 0 | 2006 | 63 | 63 | 2009 | Making a commitment to a joint responsibility for the final goals and results to be achieved |
| 2.36 | 53 | 20 | 2003 | 89 | 0 | 2005 | 64 | 47 | 2008 | Using a uniform language in the care chain |
| 2.19 | 44 | 17 | 2004 | 0 | 0 |  | 65 | 73 | 2008 | Reaching agreements on the financial budget for integrated care |
| 2.16 | 53 | 13 | 2004 | 11 | 0 | 2009 | 53 | 75 | 2009 | Allocating financial budgets for the implementation and maintenance of integrated care |
| 2.14 | 69 | 30 | 2003 | 67 | 0 | 2006 | 70 | 62 | 2009 | Involving leaders in improvement efforts in the care chain |
| 2.07 | 63 | 33 | 2004 | 78 | 0 | 2006 | 74 | 73 | 2008 | Creating an open environment that encourages experiments and pilot projects |
| 2.04 | 16 | 11 | 2005 | 0 | 0 |  | 24 | 56 | 2007 | Offering a single collaborative financial contract to the financing parties through the collective of care partners |
| **Cluster description** | | | | | | | | | | This cluster concentrates on room for innovation (experiments), leadership responsibilities for performance achievement, and joint financial agreements which cover the integrated care chain as a whole. The elements concern issues such as preconditions for entrepreneurship, including financial preconditions |
